# The Role of Angiogenesis Targeted Therapies in Metastatic Advanced Gastric Cancer: A Narrative Review

**DOI:** 10.3390/jcm12093226

**Published:** 2023-04-30

**Authors:** Izuma Nakayama, Daisuke Takahari

**Affiliations:** Department of Gastroenterological Chemotherapy, Cancer Institute Hospital of the Japanese Foundation for Cancer Research, Tokyo 135-8550, Japan; izuma.nakayama@jfcr.or.jp

**Keywords:** gastric cancer, VEGF, target therapy, clinical trial

## Abstract

Since bevacizumab was first approved by the U.S. Food and Drug Administration as an anti-angiogenic therapy in 2004, angiogenesis-targeted therapy has been developed for various types of solid tumors. To date, ramucirumab and apatinib are clinically available as treatments for metastatic advanced gastric cancer (AGC). Ramucirumab demonstrated prolonged survival as second-line therapy of metastatic AGC in the RAINBOW and REGARD trials. However, neither ramucirumab extended survival in treatment-naïve patients with AGC in the RAINFALL or RAINSTORM trials nor bevacizumab in the AVAGAST and AVATAR trials. Apatinib demonstrated superior efficacy over the best supportive care in a Chinese phase III trial but not in an international phase III (ANGEL) trial. Currently, combination therapy of ramucirumab with irinotecan or FTD/TPI is being evaluated in the third-line setting, assessing the efficacy of continuous angiogenesis inhibition from second- to third-line therapy. Recently, the role of angiogenesis inhibition via immunomodulators is attractive to clinicians. Emerging results of several early-phase clinical trials indicated the promising antitumor activity of angiogenesis inhibition in combination with immune therapy. This review offers an overview of the history of clinical trials focused on anti-angiogenic for patients with AGC and presents future perspectives in this area.

## 1. Introduction

Gastric cancer is estimated to be the fifth most common malignancy and the third leading cause of cancer-related mortality worldwide [1]. Systemic chemotherapy is the standard line of treatment for patients with gastric cancer to improve survival and quality of life [2]. However, the survival of patients with advanced gastric cancer (AGC) remains poor, with a median overall survival (OS) of approximately 15 months [3,4,5,6,7], justifying the need for new treatment options.

Molecular targeting-based therapies were investigated as a solution for the improvement of AGC patients with dismal prognoses. In 2011, the Avastin in Gastric Cancer (AVAGAST) trial was the first clinical trial to elucidate the superior efficacy of adding bevacizumab (Bev), a monoclonal antibody (mAb) against vascular endothelial growth factor (VEGF-A), in combination with chemotherapy for patients with AGC. Unfortunately, Bev did not achieve the primary endpoint [8]. In 2014, the ramucirumab plus paclitaxel versus placebo plus paclitaxel in patients with previously treated advanced gastric or gastro-oesophageal junction adenocarcinoma (RAINBOW) and ramucirumab monotherapy for previously treated advanced gastric or gastro-oesophageal junction adenocarcinoma (REGARD) trials demonstrated the effectiveness of anti-VEGF therapy in patients previously treated for AGC [9,10]. Ramucirumab (Ram), a recombinant mAb that specifically binds to VEGF-receptor 2 (VEGF-R2), inhibits the interaction of VEGFR2 with its ligands (VEGF-A, VEGF-C, and VEGF-D). Based on these results, Ram became the first targeted therapy for angiogenesis in patients with AGC [9,10].

A multikinase inhibitor with anti-angiogenic activity was expected to be a promising agent for the treatment of AGC. Although the initial therapeutic development with multikinase inhibition began with negative results, apatinib, a small-molecule tyrosine kinase inhibitor with potent suppression of VEGFR2, demonstrated efficacy in third- or later-line chemotherapy in Chinese patients with AGC [11]. Subsequently, regorafenib, an oral multikinase inhibitor targeting angiogenic (VEGFR1, VEGFR2, and tyrosine kinase with Ig and epidermal growth factor homology domain-2), stromal (platelet-derived growth factor receptor beta [PDGFRβ]), and oncogenic (rapidly accelerated fibrosarcoma [RAF], rearranged during transfection [RET], and KIT receptor tyrosine kinases, showed longer survival than placebo during salvage-line treatment of AGC outside of China [12,13].

A novel combination therapy of anti-angiogenic therapy with an immune checkpoint inhibitor has opened the door to a new era. In general, contrary to renal cancers and melanomas, gastrointestinal cancers such as AGC and colorectal cancer (CRC) are assumed to be non-immunogenic, except for subsets with microsatellite instability-high (MSI-H). A less immunogenic tumor microenvironment surrounding the AGC prevents cytotoxic lymphocytes from attacking tumor cells. Thus, anti-angiogenic agents were expected to act as immunomodulators that converted immunologically “cold” environments to “hot” ones.

In this review, we describe the progress of anti-angiogenic therapy for AGC, with particular attention to clinical trials and future perspectives of anti-angiogenic therapy for the treatment of patients with AGC.

## 2. History of Anti-Angiogenic Therapy

Neovascularization is the formation of a new functional microvascular network. Angiogenesis is a type of neovascularization by which new vessels are formed from pre-existing ones. Neovascularization is categorized into vasculogenesis, arteriogenesis, or other types. Angiogenesis is one of six known mechanisms employed by solid tumors to recruit blood vessels necessary for their initiation, growth, and metastatic spread [14].

In 1971, Folkman hypothesized that angiogenesis played a critical role in tumor enlargement beyond a few millimeters [15]. He proposed that an antibody against the tumor angiogenesis factor (TAF) secreted by tumors could potentially suppress tumor growth, which is the concept on which anti-angiogenic therapy is based. However, it took more than three decades for this concept to be proven in clinical trials [16]. At the time, the TAF factor defined by Folkman was not well understood. However, subsequent studies have identified several mediators of tumor angiogenesis and have successfully performed structural analysis to target angiogenesis. In 1983, Dvorak discovered the vascular permeability factor (VPF), a protein that increased vascular permeability, which was subsequently identified with VEGF via peptide sequencing [17,18,19]. In 1989, Leung et al. successfully purified VEGF from the medium condition by bovine pituitary folliculostellate cells [20]. Subsequent cloning of VEGF has led to the introduction of VEGF-directed therapy in clinical practice [21]. In 1993, Kim et al. reported that a VEGF-targeting mAb successfully suppressed tumor growth in vivo [22]. Following these outstanding research achievements over three decades, molecular targeted therapies for angiogenesis have been introduced to the field of cancer treatment.

### Signal Transduction via VEGF-Related Pathways

There are five structurally different types of VEGF ligands [VEGF-A, VEGF-B, VEGF-C, VEGF-D, and placental growth factor (PlGF)] and three types of receptors (VEGFR-1, VEGFR-2, and VEGFR-3) [21,23]. VEGFR-1 and -2 are expressed on endothelial cells and consist of an extracellular domain, a transmembrane region, and an intracellular tyrosine kinase domain.

When specific ligands bind to the VEGFR, signal transduction begins and activates signaling pathways downstream, resulting in increased vascular permeability, lymphangiogenesis, proliferation, survival, and migration of bone marrow endothelial cells (3). VEGF blockade inhibits these pathways, thereby affecting tumor survival, migration, and invasion.

## 3. Clinical Trials for the Treatment of Patients with AGC

### 3.1. Application of Bev in the Treatment of Patients with AGC

**Figure 1:**
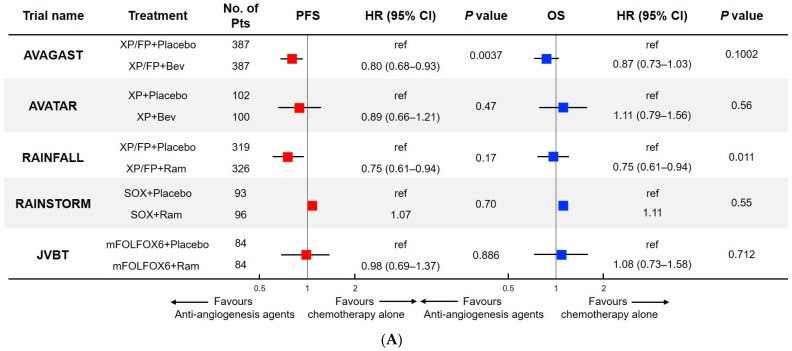

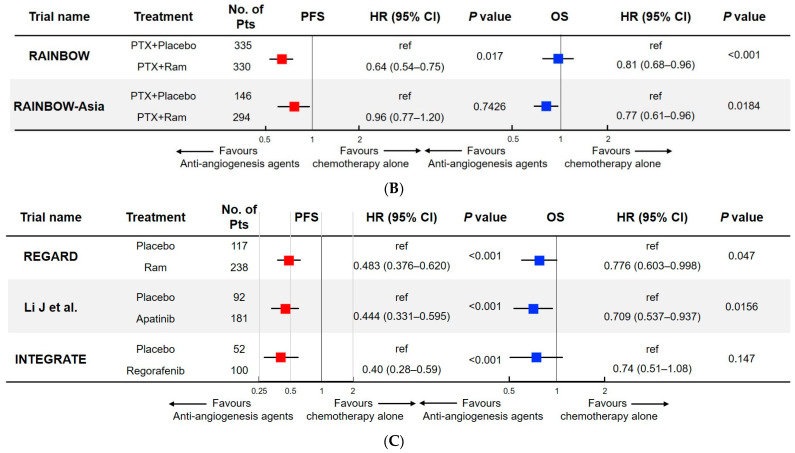
(**A**) Chemo with anti-angiogenesis agents vs. chemo alone in 1L therapy; (**B**) Chemo with anti-angiogenesis agents vs. chemo alone in 2L therapy; (**C**) Anti-angiogenesis agents vs. BSC in ≥2L therapy.

Bev demonstrated its efficacy with irinotecan (IRI), fluorouracil (FU), and leucovorin (LV) against CRC in the AVF2017g trial. Furthermore, in 2004, Bev was first approved by the U.S. Food and Drug Administration (FDA) as a molecular targeting agent for anti-angiogenic [16]. Subsequently, its efficacy has been demonstrated in non-small-cell lung cancer (NSCLC), breast cancer, ovarian cancer, cervical cancer, and malignant glioma [16,24,25,26]. Following the success of clinical trials for other malignancies, a number of clinical trials were conducted to examine the efficacy of angiogenesis therapy in the treatment of metastatic AGC. The results of these clinical trials are summarized in Table 1 and visualized in Figure 1. Shah et al. initiated a multicenter phase II study of Bev with a weekly combination of IRI and cisplatin (CDDP) as a first-line therapy for patients with AGC. They reported an overall response rate (ORR), median TTP, and median OS of 65%, 8.3 months, and 12.3 months, respectively [27]. Phase II studies of IRI plus CDDP reported an ORR, a TTP, and a median OS of approximately 50%, 5 months, and 8–9 months, respectively [28,29,30]. Considering these results, adding Bev may enhance the efficacy of standard chemotherapy for the treatment of patients with AGC as well. However, another phase II study assessing the combination of Bev with oxaliplatin (L-OHP) and docetaxel (DTX) was not as promising. This resulted in an ORR, median progression-free survival (PFS), and median OS of 42%, 6.6 months, and 11.1 months, respectively [31]. The AVAGAST trial was initiated to elucidate the add-on effect of Bev combined with chemotherapy (capecitabine [Cape] and CDDP [XP] or 5-fluorouracil [5-FU] and CDDP [FP]) as a first-line treatment for AGC [8]. A total of 774 eligible patients were randomly assigned to each treatment arm (Bev, *n* = 387; placebo, *n* = 387). Both median PFS (6.7 vs. 5.3 months; hazard ratio (HR), 0.80; 95% confidence interval (CI), 0.68–0.93; *p* = 0.0037) and ORR (46.0% vs. 37.4%; *p* = 0.0315) significantly improved with Bev, vs. placebo, respectively; however, the median OS, the primary endpoint of this study, did not (12.1 vs. 10.1 months; HR, 0.87; 95% CI, 0.73–1.03; *p* = 0.1002) [8]. The AVAGAST trial failed to demonstrate improved survival with the addition of Bev to platinum doublet chemotherapy; however, the clinical activity of Bev was statistically significant in terms of PFS and ORR. Furthermore, a pre-planned subgroup analysis indicated a survival benefit outside of Asia, especially in Latin America. The higher proportion of subsequent chemotherapy administration after progressive disease in Asia, especially in Japan, is a potential explanation for this regional difference. Another phase III randomized controlled trial (AVATAR), conducted mainly in China, evaluated the add-on effect of Bev with the XP regimen [32]. This study also failed to demonstrate any advantages in terms of the efficacy of adding Bev in the Chinese population. There were no statistically significant differences in OS, PFS, or ORR between the Bev (*n* = 100) and placebo (*n* = 102) arms. Shah et al. conducted another phase II trial to investigate the addition of Bev to the triplet regimen of DTX, CDDP, and 5-FU (DCF), which demonstrated better efficacy than FP in the V325 trial [33]. Nonetheless, due to increased toxicity from adding DTX, this triplet therapy was not accepted as the standard of care in clinical practice. Thus, they developed a modified DCF (mDCF) regimen and assessed the efficacy of adding Bev to the mDCF regimen [34]. The primary endpoint of this study was PFS at 6 months (69%; 95% CI, 63–88), surpassing the pre-planned threshold (43%). The ORR, median PFS, and OS were 67% (95% CI, 50–81), 12.0 months (95% CI, 8.8–18.2), and 16.8 months (95% CI, 12.1–26.0), respectively. This phase II study demonstrated higher ORR (34–52%) and longer PFS (7.1–8.6 months) and OS (12.6–15.1 months) than historical data of mDCF alone [35]. Furthermore, the main grade 3/4 adverse events (AEs) were neutropenia without fever (50%), venous thromboembolism (39%), and fatigue (25%). Gastrointestinal toxicity, mucositis, neuropathy, and febrile neutropenia were observed in less than 10% of patients. Promising efficacy with a manageable toxicity profile was shown; however, owing to the negative results of the AVAGAST and AVATAR trials, further evaluation of this regimen in the randomized phase III trial was not performed. However, anti-angiogenic therapy remains attractive to investigators. The challenge of seeking efficacious VEGF-targeted therapy has shifted to Ram.

### 3.2. Ram Was the First Approved Anti-Angiogenic Agent for AGC Treatment

The clinical trial using Ram for patients with AGC started in second- and later-line treatments. In the REGARD trial, the efficacy and safety of Ram monotherapy were evaluated and compared with best supportive care (BSC) alone in patients previously treated for AGC after first-line chemotherapy [10]. In total, 355 patients (238 in the Ram group and 117 in the placebo group) from 29 countries participated in the study. The median OS was 5.2 months in the Ram group and 3.8 months in the placebo group. Ram monotherapy significantly prolonged survival over BSC alone (HR, 0.776; 95% CI, 0.603–0.998; *p* = 0.047). The incidence of AEs was similar between the groups, except for hypertension (Ram: 16%, *n* = 38; placebo: 8%, *n* = 9). Severe AEs (>10%) were not observed in the Ram group. The results from the REGARD trial with Ram showed that the VEGF pathway was a valid treatment target for AGC. Thus, Ram became the first VEGF targeted agent for previously treated patients with AGC. Subsequently, the addition of Ram to standard chemotherapy agents was evaluated in the RAINBOW trial as a second-line treatment for AGC [9]. This study was conducted in 170 centers across 27 countries. Here, 665 patients enrolled and were randomly assigned to the Ram plus weekly paclitaxel (PTX) (*n* = 330) or weekly PTX (*n* = 335) groups. The median OS and PFS were 9.6 and 4.4 months in the patients who received weekly PTX plus Ram and 7.4 and 2.9 months in the patients who received weekly PTX, respectively. Statistically significant differences were observed in both OS (HR, 0.807; 95% CI, 0.678–0.962; *p* = 0.017) and PFS (HR, 0.635; 95% CI, 0.536–0.752; *p* < 0.0001). The severe AEs that occurred more frequently (>10%) in the Ram group than in the placebo group were neutropenia (41% vs. 19%), leukopenia (17% vs. 7%), and hypertension (14% vs. 2%). Currently, combination therapy with weekly PTX and Ram is recommended as the standard second-line chemotherapy for patients with AGC in the US, Europe, and East Asian countries, including Japan [36,37,38,39,40,41]. Additionally, RAINBOW-Asia was a randomized phase III study conducted predominantly in China, with a design similar to that of the RAINBOW trial [42]. In total, 440 patients were randomly assigned to the Ram (*n* = 294) and placebo (*n* = 146) groups. The co-primary endpoints were PFS and OS after protocol amendment. This study demonstrated the benefit of Ram in PFS (HR, 0.765; 95% CI, 0.613–0.955, *p* = 0.0184) but not in OS (HR, 0.963; 95% CI, 0.771–1.203, *p* = 0.7426). Here, dilution effect by use of post-discontinuation chemotherapy (Ram: 54%; placebo: 56%) was assumed to be the main reason for not observing a benefit in OS.

### 3.3. Rechallenge of Anti-VEGF Therapy in First-Line Treatment

The successful results of two global phase III trials (REGARD and RAINBOW) accelerated the conduction of first-line clinical trials to evaluate the efficacy of Ram. Thus, a phase III trial (RAINFALL) was conducted to elucidate the add-on effect of Ram with fluoropyrimidine plus CDDP as first-line chemotherapy for patients with AGC [43]. A total of 645 patients (Ram group, *n* = 326; placebo group, *n* = 319) from 126 centers in 20 countries were enrolled in this study. The primary endpoint was investigator-assessed PFS, and the secondary endpoint was OS. The median PFS of the Ram group was significantly longer than that of the placebo group (5.7 vs. 5.4 months; HR, 0.753; 95% CI, 0.607–0.935; *p* = 0.0106). The median OS in the Ram and placebo groups were 11.2 and 10.7 months, respectively (no statistically significant difference). However, there were more grade 3 hypertension and gastrointestinal perforations in the Ram group (10% and 4%, respectively) than in the placebo group (2% and 1%, respectively). The safety profile of add-on Ram was largely consistent with that observed in the previous trials. This study met its primary endpoint; however, the result was considered negative. Firstly, the difference in median PFS between the two groups was only 0.3 months, which is not clinically meaningful. Secondly, among the 458 patients for whom radiological scans were available for central review, no improvement in PFS was observed by a blinded independent central review. Thirdly, no subgroup that benefited from the addition of Ram was identified. The authors concluded that Ram in combination with cisplatin and fluoropyridine could not be recommended as a first-line treatment for AGC. Two other phase II trials of combination therapy with Ram and a platinum doublet were investigated as first-line treatments, and their results consistently supported the negative findings of the RAINFALL trial. The JVBT trial was a randomized, double-blind, phase II trial conducted at multiple centers in the US [44]. In total, 168 eligible patients were randomized to receive either modified FOLFOX6 (mFOLFOX6) plus Ram or mFOLFOX6 plus placebo. This study did not meet the primary endpoint, PFS (stratified HR, 0.98; 95% CI, 0.69–1.37; *p* = 0.886), or the secondary endpoint, OS (stratified HR, 1.08; 95% CI, 0.73–1.58; *p* = 0.712). Furthermore, an enhancement in ORR was not reported (Ram: 45.2%; placebo: 46.4%). Another attempt to investigate the efficacy of Ram as a first-line treatment was initiated in an East Asian cohort [45]. The RAINSTORM trial was a randomized, double-blind, phase II trial conducted at 36 sites in Japan, South Korea, and Taiwan. This study primarily aimed to evaluate the add-on effect of Ram with S-1 plus L-OHP (SOX) in a first-line setting and the continuous use of Ram from first-to second-line treatment. Eligible patients were randomly assigned to receive Ram plus SOX or placebo plus SOX in Part A. After discontinuation of first-line therapy, all eligible patients received Ram plus PTX as protocol treatment in Part B. Based on the findings of the previous phase Ib trial (JVCX), Ram was administered on days 1 and 8 of a 21-day cycle to maintain a higher trough level [46]. The primary endpoint was PFS after first-line treatment. In total, 191 patients were randomized to the Ram with SOX (*n* = 96) or placebo plus SOX (*n* = 93) groups. Median PFS was 6.34 and 6.74 months in the Ram and placebo groups, respectively, demonstrating no statistically significant difference (HR, 1.07; 80% CI, 0.86–1.33; *p* = 0.70). The median OS was similar between the two groups (Ram: 14.65; placebo: 14.26 months in the placebo group). No new safety signals were observed.

Ram, with a more potent anti-angiogenic effect than Bev, did not demonstrate improved survival in previously untreated patients with AGC, regardless of backbone chemotherapy regimens or administration schedules. The effect of post-discontinuation therapy was not consistently observed in the RAINFALL subgroup analysis. Furthermore, the negative results were commonly demonstrated in the two phase II trials conducted in the USA and East Asia [43,44,45]. Plasma VEGF-A levels were a promising biomarker for predicting anti-VEGF-directed therapy in the AVAGAST trial [47]. However, this was not validated in the RAINFALL trial [43]. Additionally, ziv-aflibercept (AFL), a recombinant fusion protein that functions as a soluble decoy receptor binding to VEGF-A, VEGF-B, and PlGF, with FOLFIRI demonstrated better efficacy than FOLFIRI alone in metastatic colorectal cancer (mCRC) [48]. However, the randomized phase II (ZEMEGA) trial evaluated the safety and efficacy of adding AFL to mFOLFOX6 and did not demonstrate superiority in terms of PFS at 6 months (primary endpoint) as a first-line treatment for metastatic esophagogastric adenocarcinoma [49].

In conclusion, these attempts lasting over a decade showed that anti-VEGF antibodies could not improve the survival of patients with AGC in a first-line setting. Also, no robust predictive biomarker has been identified yet. However, the ARMANI trial (NCT03223376), an ongoing phase III trial comparing switch maintenance therapy with PTX plus Ram and first-line continuation, is evaluating the potential of anti-VEGF-directed therapy [50].

### 3.4. Combination Therapy with Ram and Nanoparticle Albumin-Bound (nab)-PTX

Since Ram with PTX became the standard of care for patients with AGC after the RAINBOW and REGARD trials, the search for a better partner for Ram has been explored in several more recent trials.

Nab-PTX is a solvent-free nanoparticle formulation of PTX that minimizes the risk of hypersensitivity to polyethoxylated castor oil and hydrated ethanol, without steroid premedication. The non-inferiority of weekly nab-PTX to weekly PTX was shown in the ABSOLUTE trial, an open-label randomized non-inferiority phase III trial [51]. Currently, nab-PTX is available in Japan as a treatment option for patients who are intolerant to alcohol, have uncontrollable diabetes mellitus, or have previously experienced hypersensitivity to PTX [40]. Following the ABSOLUTE trial, a phase II trial of nab-PTX in combination with Ram was conducted in patients with previously treated AGC [52]. The ORR, assessed by an independent review committee, was the primary endpoint of this study. PFS and OS were secondary endpoints. A total of 45 patients enrolled, 43 of whom received the study treatment. Notably, ORR was 54.8% (90% CI, 41.0–68.0). The median PFS was 7.6 months (95% CI, 5.4–8.1) and OS did not reach the cut-off date. Decreased neutrophil count was the most common grade 3/4 AE (76.7%). Still, febrile neutropenia was observed in two patients (4.7%). Considering the ORR of PTX with Ram (28%) in the RAINBOW trial, combination therapy with nab-PTX and Ram demonstrated promising activity with high ORR and manageable toxicity. However, the ORR of combination therapy of Ram and nab-PTX was found to be 15% (CI 95%, 6.6–24.2) in another phase II study [53]. However, the efficacy of Ram in combination with nab-PTX was not validated. Subgroup analysis of the ABSOLUTE trial showed weekly nab-PTX to be more efficacious than weekly PTX in the subset with a larger amount of ascites [51]. Afterward, a post-hoc exploratory analysis of the ABSOLUTE trial was conducted to assess whether nab-PTX had better efficacy than PTX in patients with apparent peritoneal metastasis (PM) [54]. In the PM group, the median OS with nab-PTX and Ram were 9.9 and 8.7 months, respectively. Nab-PTX showed significantly longer OS (HR, 0.63; 95% CI, 0.45–0.88, *p* = 0.0060). Whereas, in the no-PM group, a shorter median OS of nab-PTX (11.6 months) than that of PTX (15.7 months) was evident (HR, 1.40; 95% CI, 1.06–1.86; *p* = 0.0180). A phase II trial (*p*-SELECT, WJOG10617G) was conducted to prospectively compare the efficacy of PTX plus Ram with that of nab-PTX plus Ram in patients with AGC with peritoneal dissemination refractory to first-line therapy [55]. The median OS of nab-PTX plus Ram and PTX plus Ram were 8.1 months (95% CI, 6.4–10.3) and 7.2 months (95% CI, 5.6–11.5), respectively, showing no significant difference (HR, 0.96; 95% CI, 0.62–1.48; *p* = 0.63) [56]. Thus, there is no consensus on the preferred use of nab-PTX plus Ram over PTX plus Ram for AGC patients with PM.

### 3.5. Combination Therapy with IRI

IRI monotherapy is a therapeutic option in the second or later lines of chemotherapy for patients with AGC [57,58,59,60,61,62]. FOLFIRI plus Ram is the standard second-line treatment for metastatic CRC [63]. A new combination of Ram with IRI could be an alternative treatment to PTX-based regimens for patients with neurotoxicities induced by L-OHP or early recurrence after docetaxel-containing regimens, such as S-1 plus DTX (DS), 5-FU, LV, L-OHP, and DTX (FLOT). The RAMIRIS study was a randomized phase II trial that compared the efficacy of FOLFIRI plus Ram with PTX plus Ram as second-line treatments for AGC [64]. The primary endpoint was the proportion of OS rate at 6 months. In total, 111 patients (FOLFIRI + Ram, *n* = 72; PTX + Ram, *n* = 38) were enrolled. The estimated OS rate at 6 months in the FOLFIRI plus Ram group was 54% (95% CI, 44–67), which was below the anticipated level (65%).

The primary endpoint was not met in this study. However, in the pre-planned subgroup analysis of prior to receiving DTX, longer OS was observed in the FOLFIRI plus Ram group (7.5 months) than in the PTX plus Ram group (6.6 months), without statistically significant differences (HR, 0.81; 95% CI, 0.47–1.43). The ORR and PFS of FOLFIRI plus Ram were more favorable than those of PTX plus Ram in DTX-pretreated patients. The proportions of both all-grade and severe-grade AEs were similar between the two arms, but different toxicity profiles were observed. Hematological and gastrointestinal toxicities mainly occurred with FOLFIRI plus Ram. However, peripheral neuropathy was more common with PTX plus Ram than with FOLFIRI plus Ram (47% vs. 21%). Two single-arm trials of combination therapy with IRI and Ram have been conducted in Japan. One was a phase Ib study to determine the recommended dose (RD) of IRI in combination with Ram in the Japanese population [65]. Six patients with previously treated AGC participated in this study. The RD IRI was 150 mg/m^2^ in combination with Ram. No dose-limiting toxicity was observed. One of the six patients achieved a partial response. The HGCSG1603 trial was a phase II study that evaluated the efficacy of IRI (150 mf/m^2^) plus Ram as a second-line therapy for patients with AGC [66]. Patients harboring homozygous or double heterozygous UGT1A1*6 or UGT1A1*28 received a reduced IRI dose (120 mg/m^2^). The primary endpoint was PFS rate at 6 months. Thirty-five patients enrolled in this study. As first-line therapy, 32 patients (91.4%) received platinum-based doublet chemotherapy (oxaliplatin, *n* = 31; CDDP, *n* = 1) and three received taxane-containing regimens. The PFS rate at 6 months was 26.5% (95% CI, 13.2%–41.8%; *p* = 0.1353), not achieving its primary endpoint. The median PFS was 4.2 months (95% CI, 2.5–5.4) and the median OS was 9.6 months (95% CI, 6.4–16.6). The ORR was 25.9% (95% CI, 11.1%–36.3%). The authors concluded that this new combination of IRI and Ram exhibited antitumor activity and manageable toxicity.

No successful results of Ram combined with an IRI-based regimen have been reported as a second-line treatment for AGC. However, the aforementioned trials provided insight that this combination might be an alternative treatment option to DTX pretreatment for patients with progressive disease or with persistent neuropathy induced by L-OHP during adjuvant or first-line chemotherapy.OGSG1901 study is now ongoing, which evaluate the efficacy of IRI plus Ram for the AGC patients with early relapse during or after adjuvant DTX plus S-1 [67].

Currently, a phase III trial (RINDBeRG UMIN000023065) of Ram plus IRI as third- or later-line treatment beyond progression after Ram for patients with AGC has completed enrollment and is awaiting analysis of its results. In the ML18147 trial for patients with mCRC, the continuation of Bev beyond first-line progression, called Bev beyond progression (BBP), succeeded in prolonging patient survival [68]. The RINDBeRG trial aimed to evaluate the add-on effect of Ram on IRI and persistent VEGF suppression by switching backbone chemotherapy.

### 3.6. Combination Therapy with Trifluridine/Tipiracil (FTD/TPI)

FTD/TPI is an oral cytotoxic agent consisting of a thymidine-based nucleoside analog, trifluridine, and a thymidine phosphorylase inhibitor, tipiracil. Based on the positive results of the TAGS trial, FTD/TPI is one of the standard therapies for third- or later-line patients with AGC [69]. Promising antitumor activity of FTD/TPI with Bev has been reported for the treatment of mCRC [70,71,72]. Moreover, based on the mCRC findings, a Danish (LonGas) trial was conducted evaluating the addition of Bev on FTD/TPI in a randomized phase II design. A total of 103 patients randomly received FTD/TPI with or without Bev. There was no improvement in PFS (median 3.9 vs. 3.7 months, HR 0.74, *p* = 0.14), OS (median 9.6 vs. 9.0 months, HR 0.91, *p* =0.67), or ORR (6% vs. 2%, *p* = 0.35). Subgroup analysis results showed the benefit of adding Bev to the third-line treatment [73]. In Japan, a single-arm, two-cohort, phase II study exploring the safety and clinical activity of FTD/TPI combination therapy and Ram was conducted [74]. This study included two cohorts: cohort A (Ram-naïve patients) and cohort B (patients previously treated with Ram). A total of 64 patients were enrolled, of whom 33 were included in cohort A as second-line therapy and 31 in cohort B as third- or later-line therapy. The disease control rate (DCR), determined by the investigator’s assessment of each cohort, was adopted as the primary endpoint. The DCRs of cohorts A and B were 85% (95% CI, 68–95) and 77% (95% CI, 59–90), respectively, while the ORRs were 9% (95% CI, 2–24) and 16% (95% CI, 6–34), respectively. Tumor shrinkage from baseline was observed in 22 patients (71%) in cohort A and 21 patients (68%) in cohort B. Remarkably, increases in ORR were observed in patients who had previously received immunotherapy compared to those who had not. In cohort A, the ORR was 29% in patients with prior immunotherapy (95% CI, 4–71) and 4% in patients without (95% CI, 0–20), whereas in cohort B, the ORR was 33% (95% CI, 12–62). Treatment-related AEs demonstrated similar frequency between the two cohorts (cohort A: 97%; cohort B: 100%) but were tolerated well. Decreased neutrophil count was the most common grade 3/4 AE (cohort A: 82%; cohort B: 74%), which was much higher than in the patients with FTD/TPI in the TAGS trial (34%) [69]. However, febrile neutropenia was rarely seen in either cohort (3% in both). Another phase II study including FTD/TPI plus Ram was conducted at a single institute in the US. Here, a DCR rate of 88% was reported, similar to that observed in the Japanese trial. Currently, a randomized phase II study (WJOG15822G) comparing the safety and efficacy of FTD/TPI with and without Ram as a third-line or later therapy is ongoing in Japan. Moreover, in the US, a randomized phase II clinical trial comparing RAM with FTD/TPI and PTX in a second-line therapy setting is ongoing (NCT04660760).

### 3.7. Multikinase Inhibitors

Kinase inhibitors targeting angiogenesis, such as sorafenib and sunitinib, are effective against renal cell carcinoma (RCC) and hepatocellular carcinoma (HCC) [75,76,77]. Several clinical trials have revealed that intratumor heterogeneity in AGC represents a significant obstacle to the effectiveness of molecular targeting agents [78]. However, multikinase inhibition using small molecules can simultaneously suppress multiple pathways related to the proliferation or survival of tumor cells and is expected to overcome intratumor heterogeneity [79]. Since the 2010s, several attempts have been made to treat AGC using multikinase inhibitor monotherapy or combination with chemotherapy [11,80,81,82,83,84,85,86,87,88,89,90]. However, only apatinib and regorafenib showed positive results in a phase III trial to date [11,13].

A randomized phase II trial of apatinib was conducted in China in patients with heavily pretreated AGC [91]. PFS was set as the primary endpoint of this study. Both apatinib (850 mg once daily) and apatinib (425 mg twice daily) showed superior PFS and OS over placebo. No statistically significant differences were observed in the different treatment schedules. However, severe grade hand-foot syndrome (4.26% vs. 13.04%) and hypertension (8.51% vs. 10.87%) occurred more frequently with apatinib. Consequently, apatinib was recommended, and further investigations were performed in a phase III trial. A randomized, double-blind, placebo-controlled phase III trial of apatinib was performed in 32 centers in China on patients with AGC refractory to chemotherapy [11]. The OS and PFS were defined as dual primary endpoints. A total of 267 patients were randomized and assigned to the apatinib (*n* = 176) or placebo (*n* = 91) groups in the full analysis set. All patients previously received more than two lines of chemotherapy, and approximately 35% of patients received apatinib (34.1%) or placebo (36.3%) as the fourth- or later-line. Median OS were 6.5 months (95% CI, 4.8–7.6) and 4.7 months (95% CI, 3.6–5.4), while median PFS were 2.6 months (95% CI, 2.0–2.9) and 1.8 months (95% CI, 1.4–1.9) in the apatinib and placebo groups, respectively. Apatinib demonstrated a significant improvement in survival (HR, 0.709; 95% CI, 0.537–0.937; *p* = 0.0156) compared to the placebo. Apatinib also demonstrated improved PFS (HR, 0.444; 95% CI, 0.331–0.595, *p* < 0.001). Grade 3/4 hypertension (4.5% vs. 0.0%), proteinuria (2.3% vs. 0.0%), and hand-foot syndrome (8.5% vs. 0.0%) occurred more frequently in the apatinib than in the placebo group. In 2014, apatinib was approved by the China Food and Drug Administration as a third-line chemotherapy for AGC. However, prolonged OS was not demonstrated in a global randomized phase III (ANGEL) trial conducted outside China [92]. Currently, three pivotal phase III trials of apatinib for patients with AGC are ongoing in China. Two trials are evaluating the efficacy and safety of combination therapy with immune checkpoint inhibitors (SHR-1210 in NCT04342910; camrelizumab in NCT04342910). The third is comparing the maintenance with capecitabine plus apatinib and observation after initial treatment (NCT03889626).

Regorafenib was the first multikinase inhibitor to demonstrate a survival benefit in a global phase III trial in patients with AGC. Regorafenib is an orally administered multikinase inhibitor that targets several kinases involved in the regulation of tumor angiogenesis (VEGFR1, VEGFR2, VEGFR3, and TIE2), oncogenesis (KIT, RET, and RAF), and the tumor microenvironment (PDGFR and FGFR) [93]. A randomized, placebo-controlled, phase III trial (CORRECT) showed the activity of regorafenib with a survival benefit in the salvage line of mCRC treatment [94]. An international double-blind phase II trial (INTEGRATE) was conducted in Australia, New Zealand, South Korea, and Canada to explore the clinical activity and safety of regorafenib in patients previously treated for AGC [12]. A total of 147 patients were evaluated; 97 patients were assigned to receive regorafenib (160 mg, once daily) and 50 patients to a placebo. The primary endpoint, median PFS, of the regorafenib group (2.6 months; 95% CI, 1.8–3.1) was significantly longer than that of the placebo group (0.9 months; 95% CI, 0.9–0.9) (HR, 0.40; 95% CI, 0.28–0.59; *p* < 0.001). The OS had a trend in favor of regorafenib (HR, 0.74; 95% CI, 0.51–1.08; *p* = 0.147). Following these favorable results, a randomized phase III study (INTEGRATE II) of regorafenib versus placebo as third- or later-line treatment in refractory AGC was initiated. The INTEGRATE II trial was amended to divide the cohort into INTEGRATE IIa (regorafenib alone vs. placebo) and IIb (regorafenib plus nivolumab (Nivo) vs. placebo). Recently, the INTEGRATE IIa results were reported [95]. A total of 251 participants from six countries (Australia, South Korea, Japan, Taiwan, New Zealand, and Canada) were randomized to receive regorafenib (*n* = 169) or a placebo (*n* = 82). Regorafenib resulted in the prolongation of OS, the primary endpoint (HR, 0.68; 95% CI, 0.52–0.90; *p* = 0.006), and improved PFS (HR, 0.53; 95% CI, 0.40–0.70; *p* < 0.001), compared to the placebo. Furthermore, the combined data of INTEGRATE and INTEGRATE IIa trials demonstrated improved OS (HR, 0.70; 95% CI, 0.56–0.87; *p* = 0.001), compared to the placebo. The positive data for apatinib were limited in China. However, the clinical activity of apatinib in the ORR and PFS was consistently observed in the global ANGEL study.

Thus, these results, together with those of the INTEGRATE study, indicate that multikinase inhibitors may be effective in the treatment of AGC. However, the disappointing results of other multikinase inhibitors suggest that these inhibitors alone may only offer a moderate clinical benefit over no treatment.

## 4. Anti-Angiogenic Therapy with Immunotherapy

Several preclinical studies have reported that anti-angiogenic agents have the potential to convert unfavorable immune microenvironments into favorable ones by reducing the number of tumor-associated macrophages and preventing CD8+ T-cells from infiltrating tumor cells [96]. Herein, we review recent early-phase clinical trials related to novel combination therapies involving anti-angiogenic agents and immunotherapy.

### 4.1. Ramucirumab Plus Anti-PD1/PD-L1 Therapy without Cytotoxic Agents

Simultaneous blockade with anti-VEGF-directed therapy and anti-PD-1/PD-L1 has demonstrated efficacy in treating metastatic NSCLC and RCC [97,98,99,100]. Following these successful results, dose escalation and expansion studies have been considered in the treatment of patients with AGC (Table 2). The JVDF study examining the safety of combination therapy of Ram (8 mg/kg) and pembrolizumab (Pembro) (200 mg), an anti-PD-1 antibody, was conducted at multiple centers in the US, France, Germany, Spain, and the UK [101]. A total of 41 previously treated patients with AGC were enrolled. One patient died, possibly because of disease progression. The standard dose and schedule were safe and feasible for this novel combination. The ORR and DCR were 7% (95% CI, 1.5–19.9) and 51% (95% CI, 35.1–67.1), respectively. Median PFS and OS were 2.5 (95% CI, 1.5–4.2) and 5.9 (95% CI, 4.4–10.6), respectively. The cohort was expanded with an additional 28 treatment-naïve patients [102]. The clinical outcomes were as follows: ORR, 25%; median PFS, 5.6 months; OS, 14.6 months. Enhanced antitumor activity occurred in patients with high PD-L1 expression (combined positive score (CPS ≥ 1). The ORR was 32% (CPS ≥ 1) vs. 17% (CPS < 1); median PFS, 8.6 months (CPS ≥ 1) vs. 4.3 months (CPS < 1); and OS, 17.3 months (CPS ≥ 1) vs. 11.3 months (CPS < 1). The JVDJ study was similarly designed as a multi-cohort phase Ia/Ib study of Ram (8 mg/kg) with durvalumab (750 mg), an anti-PD-L1 mAb, and was conducted at 25 centers in eight countries [103]. Overall, 29 patients with previously treated AGC were included. The ORR was 21%; DCR, 55%; median PFS, 2.6 months (95% CI, 1.5–7.1); and OS, 12.4 months (95% CI, 5.5–16.9). The only grade 3/4 AEs occurring in >10% of patients were hypertension (17.2%) and pulmonary embolism (10.3%). In Japan, the NivoRam study was conducted to assess the feasibility of administering Nivo (3 mg/kg) and Ram (8 mg/kg) to patients with AGC after first-line disease progression [104]. The primary endpoint, PFS at 6 months, was 37.4% (90% CI, 25.7–49.2). The ORR was 26.7%; DCR, 62.2%; median PFS, 2.9 months; and OS, 9.0 months. Given that the ORR and DCR of Nivo alone were 11.2% and 40.3% in the Attraction-2 study, respectively [105], this novel combination therapy has the potential to enhance antitumor activity. Notably, the combination of Ram and anti-PD-1/PD-L1 consistently demonstrated tolerable toxicity without dose reduction of individual agents.

### 4.2. Multikinase Inhibitor with Anti-PD-1 Therapy without Cytotoxic Agents

An exploratory analysis of apatinib with SHR1210, an anti-PD-1 antibody, was conducted in China in patients with advanced HCC or AGC [106]. This study was designed as a dose-escalation study of once-daily oral apatinib at 125, 250, or 500 mg in combination with SHR1210 at 200 mg, every two weeks. Among the five patients (AGC, *n* = 3; HCC, *n* = 2) receiving 250 mg apatinib, only one asymptomatic dose-limiting toxicity (DLT) (increased lipase) was observed. Five patients (AGC, *n* = 3 and HCC, *n* = 2) reached apatinib 500 mg in the dose-escalation cohort; however, severe hypertension and immune-related pneumonia occurred in two (40%) and three (60%) patients, respectively. The RD was set at 250 mg apatinib. Among the 23 evaluable patients with AGC, the ORR was 17.4% (95% CI, 5.0–38.9%); DCR, 78.3% (95% CI, 56.3–92.5%); median PFS, 2.9 months (95% CI, 2.5–4.2); and median OS, 11.4 months (95% CI, 8.6–NA). Among the 33 patients receiving apatinib 250 mg, grade 3/4 AEs (>10%) were hypertension (*n* = 5, 15.2%) and increased aspartate aminotransferase (AST) levels (*n* = 5, 15.2%). Combination therapy with 250 mg apatinib and SHR-1210 demonstrated a moderate increase in efficacy with tolerable toxicity. Based on the findings of regorafenib-induced immunomodulation in preclinical models [107,108], the REGONIVO study, an open-label, dose-escalation, and dose-expansion phase Ib trial, was conducted on previously treated patients with AGC and CRC [109]. Patients received regorafenib 80–160 mg once daily for 21 of the 28 days and 3 mg/kg Nivo every 2 weeks. A total of 25 patients with AGC, all of whom had previously received two or more lines of chemotherapy, were included. Among them, 23 (92%) had received prior anti-angiogenic therapy and 7 (28%), immunotherapy with anti-PD-1/PD-L1 antibodies. None of the patients with MSI-H were included in the AGC cohort. Once 120 mg regorafenib was determined as the maximum tolerated dose (MTD), an additional 18 patients received 120 mg regorafenib in the dose expansion cohort (AGC, *n* = 11; mCRC, *n* = 7). However, mainly due to skin toxicity, 21/25 patients (84%) required dose reduction from 120 to 80 mg. Ultimately, 80 mg regorafenib was determined as the RD in combination with Nivo. The ORR of the patients with AGC was 44% (95% CI, 24.4–65.1). Notably, of the seven patients refractory to anti-PD1/PD-L1 therapy, three achieved a partial response. Median PFS and OS were 5.6 months (95% CI, 2.7–10.4) and 12.3 months (95% CI, 5.3-NR), respectively. The most common grade ≥3 AEs of 80 mg regorafenib were hypertension (9%, *n* = 2), proteinuria (9%, *n* = 2), and liver dysfunction (9%, *n* = 2). The ORR in this study demonstrated the synergistic effect of this novel combination, considering the lower ORR of Nivo or regorafenib alone [12,105]. The clinical outcomes of this study were attractive compared to those of previous trials on the salvage line of AGC [58,61,62,69]. However, these results were not reproduced in the other cohorts. The REGOMUNE study, a phase II study of regorafenib and avelumab, an anti-PD-L1 monoclonal antibody, showed an ORR of 16.7%, a median PFS of 1.9 months, and a median OS of 7.5 months in the second- or later-line treatment of immunotherapy-naïve patients with ACG [110]. Currently, a randomized phase III (INTEGRATE IIb) trial is ongoing to evaluate the safety and efficacy of regorafenib plus nivolumab versus standard chemotherapy (PTX, DTX, IRI, or FTD/TPI) as third- or later-line therapy (NCT04879368).

### 4.3. Ram Plus Chemotherapy with Immunotherapy

Another phase I/II multicenter study of Nivo/Ram with PTX for patients with previously treated AGC was conducted in Japan. Patients initially received the standard dose and schedule of Nivo (3 mg/kg, days 1 and 15), Ram (8 mg/kg, days 1 and 15), and PTX (80 mg/m^2^, days 1, 8, and 15). A total of 43 patients (6 in phase I and 37 in phase II) enrolled. DLT was observed in two patients (one with febrile neutropenia and one with neutropenia lasting >8 days) in phase I, and RD was determined at the standard dose. The primary endpoint, PFS at 6 months, was 46.5% (80% CI, 36.4–55.8, *p* = 0.067), exceeding the predefined threshold (35%). Median PFS was 5.1 months (95% CI, 4.5–6.5); median OS, 13.1 months (95% CI, 8.0–16.6); ORR, 37.2% (95% CI, 23.0–53.5); and DCR, 83.7% (95% CI, 69.3–93.2). Given that the subgroup analysis of the RAINBOW trial reported that ORR and DCR in Japanese patients receiving PTX plus Ram were 41.2% and 94.1%, respectively, enhancement of efficacy by adding Nivo seemed not clear in this phase II trial [111]. Still, similar trials are ongoing (NCT0 4069273: PTX plus Ram with pembrolizumab) and (NCT03966118: PTX plus Ram with avelumab).

### 4.4. Therapeutic Development of Multikinase Inhibitors and Immunotherapy in the Frontline

Based on the success of the CheckMate-649 and Attraction-4 trials, first-line Nivo with chemotherapy is the standard of care for HER2-negative patients with AGC at the moment, especially with PD-L1 CPS ≥5 [6,7]. Currently, the synergistic enhancement of immunotherapy with anti-angiogenic agents would be beneficial in frontline healthcare. A phase II (EPOC1706) trial of lenvatinib (LEN), an orally administered multikinase inhibitor targeting VEGFR1-3, FGFR1-4, PDGFRα, RET, KIT, and Pembro was conducted on patients with AGC in a single Japanese institute, irrespective of the treatment line [112]. Although there had been no prospective clinical trials of a combination of LEN (20 mg once daily) and Pembro (200 mg, once every three weeks) for patients with AGC at that time, the safety data with promising efficacy for other solid tumors encouraged the initiation of this study [113]. The primary endpoint was investigator-assessed ORR. Among all patients, 14 (48%) received treatment as first-line and 15 (52%) as second-line. Patients with deficient mismatch repair (dMMR) tumors (2 patients [7%]) and PD-L1 CPS ≥ 10 (5 patients [17%]) were included. Notably, the ORR was 69% (95% CI, 49–85) and DCR, 100% (95% CI, 88–100). Furthermore, favorable antitumor activity was 70% (95% CI, 50–86) and was maintained in all patients except for two with dMMR. Although this study was a single-arm study with a limited number of patients, Pembro with LEN without chemotherapy reported much greater antitumor activity than first-line Pembro alone in the KEYNOTE-062 (14.8%, in CPS > 1) and KEYNOTE-061 (16% in CPS > 1) trials, and Pembro with Ram (25% in all and 32% in CPS ≥ 1) [102,114,115]. No new safety signals were observed, and no patient discontinued treatment owing to AEs. Validation in an ongoing phase II study of Pembro and LEN is awaiting (NCT03321630, 05041153). Meanwhile, a phase III study to elucidate the safety and efficacy of LEN plus Pembro with chemotherapy as a first-line therapy for advanced or metastatic G/GEJ adenocarcinoma is ongoing (NCT04662710).

## 5. Conclusions and Future Perspectives

**Figure 2:**
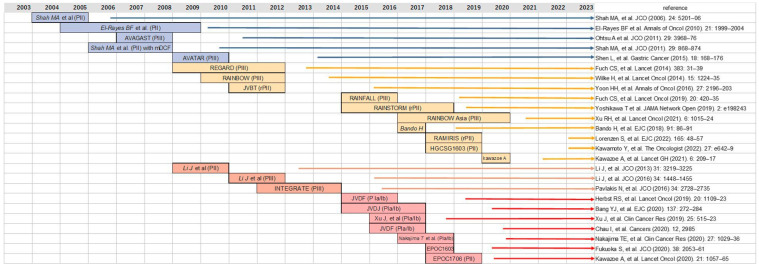
An overview of the clinical trials examining anti-angiogenic therapy in metastatic AGC. The years in which clinical trials were conducted are indicated by bands. The arrows denote the time from the papers’ publication to the present. Blue arrows represent bevacizumab, yellow ramucirumab, orange apatinib and regorafenib, and red combination therapy of anti-angiogenic therapy with immunotherapy [8,9,10,11,12,27,31,32,34,42,43,44,45,52,64,66,74,91,101,102,103,106,109,112,119].

In this review, we evaluated the progress of targeted therapy against angiogenesis for the treatment of AGC (Figure 2). Although anti-angiogenic therapies for patients with AGC have been tested in clinical trials over the last two decades, only Ram with or without PTX, and apatinib (only in China) have been established as the standard of care after second- and third-line therapy, respectively. At the initial stages of molecular targeted therapy, anti-angiogenic therapy was expected to play a critical role in clinical oncology. However, Bev did not improve the survival of previously untreated patients with AGC. The success of Ram in the RAINBOW and REGARD trials seemed promising; however, Ram did not demonstrate survival benefits in a first-line therapy setting. Although the addition of anti-angiogenic agents consistently demonstrated an enhancement in ORR and PFS, these could not be translated into survival benefits in the first-line treatment of patients with AGC [8,43,45]. These results imply that anti-angiogenic agents do not have a strong effect on the prolongation of survival but work as chemosenstizer for combination cancer therapy [116]. The post-progression therapy might dilute the efficacy of anti-angiogenic therapy provided as a prior-line therapy. The promising results of multikinase inhibitors (targeting angiogenesis), such as apatinib and regorafenib, in salvage line treatment were in line with the interpretation mentioned above. The efficacy of either apatinib or regorafenib was slightly superior as compared to no treatment. However, the multikinase inhibitor alone was unable to show survival benefit in the front-line treatment of patients with AGC who have other therapeutic options after discontinuation.

Efficacy of continuous angiogenesis inhibition beyond disease progression is a matter of interest in assessing the role of anti-angiogenic agents in combination with chemotherapy for patients with AGC. Once efficacy of Bev beyond progression was confirmed in the ML18147 trial, it was discovered that it affected the entire treatment sequence in mCRC [68], and persistent anti-angiogenesis with switching of the backbone chemotherapy became the standard of care [117]. The upcoming result of the RINDBeRG trial may potentially change the concept of AGC treatment. A similar concept study is ongoing in combination with FTD/TPI and Ram (WJOG15822G).

Furthermore, although several attempts have been made to explore the predictive markers for angiogenesis, no biomarker for routine clinical use has been identified to pinpoint which patients are most likely to benefit from the treatment [47,118]. Anti-angiogenic agents mainly affect the tumor microenvironment. Moreover, racial or sexual differences are expected to influence angiogenetic reactions. A comprehensive understanding of the tumor microenvironment and interactions between the tumor and surrounding cells is critical for incorporating personalized medicine in angiogenesis therapy.

To date, anti-angiogenic therapy has not demonstrated marked efficacy in revolutionizing AGC treatment. However, these historic molecular agents have played an essential role as by-players in immune-oncology. Ongoing clinical trials investigating the combination of anti-angiogenic agents with immunotherapy approaches may establish a novel role for anti-angiogenic agents as immunomodulators in the treatment of AGC (Table 3). First generation of immuno-oncology revolutionized cancer care by immune checkpoint blockade in intrinsically immunogenic tumors, such as melanoma RCC or NSCLC. However, durable responders of immune checkpoint blockade alone remain limited in patients with AGC. Vascular normalization with anti-angiogenic agents could contribute toward the conversion of the immunologically suppressive tumor microenvironment to a supportive microenvironment. Currently, researchers are exploring optimal partner agents and timing for multikinase inhibitors, indicating that angiogenesis therapy has paved the way for the next phase of treatments.

## Figures and Tables

**Table 1:** Clinical trials with anti-angiogenic agents and chemotherapy.

Trial Name/Authors	Ph	Regimen	Line	No. of Patients	Primary Endpoint	MST (95% CI) (Months)	HR (95% CI) *p*-Value	mPFS (95% CI) (Months)	HR (95% CI) *p*-Value	ORR (95% CI) (%)	OR (95% CI) *p*-Value	
**Bevacizumab (Bev)**												
Shah MA, et al.	II	Bev (15mg/kg) +IRI + CDDP	1L	47	TTP	12.3 (11.3–17.2)		8.3 (5.5–9.9) *TTP		65 (46–80)		
El-Rayes BF, et al.	II	Bev (7.5mg/kg) +DTX + L-OHP	1L	38	PFS	11.1 (8.2–15.3)		6.6 (4.4–10.5)		42 (28–58)		
AVAGAST	III	Bev (7.5mg/kg) +XP or FP vs. Placebo + XP or FP	1L	387 (Bev) vs. 387 (PBO)	OS	12.1 (11.1–13.8) vs. 10.1 (9.0–11.3)	0.87 (0.73–1.03) *p* = 0.1002	6.7 (5.9–7.1) vs. 5.3 (4.4–5.6)	0.80 (0.68–0.93) *p* = 0.0037	46.0 vs. 37.4	*p* = 0.315	negative
AVATAR	III	Bev (7.5 mg/kg) +XP vs. Placebo + XP	1L	100 (Bev) vs. 102 (PBO)	OS	11.4 (8.6–16.0) vs. 10.5 (8.9–14.1)	1.11 (0.79–1.56) *p* = 0.56	6.0 (4.9–7.4) vs. 6.3 (5.7–7.4)	0.89 (0.66–1.21) *p* = 0.47	41 vs. 34	1.19 (0.65–2.20) *p* = 0.35	negative
Shah MA, et al.	II	Bev (10 mg/kg) + mDCF	1L	44	PFS at 6 m	12.0 (8.8–18.2)		16.8 (12.1–26.0)		67 (50–81)		positive
**Ramucirumab (Ram)**												
RAGERD	III	Ram vs. Placebo	2L	238 (Ram) vs. 117 (PBO)	OS	5.2 (IQR 2.3–9.9) vs. 3.8 (IQR 1.7–7.1)	0.78 (0.60–0.99) *p* = 0.047	2.1 (IQR 5.9–7.1) vs. 1.3 (IQR 1.3–4.2)				positive
RAINBOW	III	Ram + PTX vs. Placebo + PTX	2L	330 (Ram) vs. 335 (PBO)	OS	9.6 (8.5–10.8) vs. 7.4 (6.3–8.4)	0.81 (0.68–0.96) *p* = 0.017	4.4 (4.2–5.3) vs. 2.9 (2.8–3.0)	0.64 (0.54–0.75) *p* < 0.001	28 (23–33) vs. 16 (13–20)	*p* = 0.315	positive
RAINBOW-Asia	III	Ram + PTX vs. Placebo + PTX	2L	294 (Ram) vs. 146 (PBO)	OS/PFS	8.7 (8.0–9.5) vs. 7.9 (6.3–9.1)	0.96 (0.77–1.20) *p* = 0.7426	4.1 (3.7–4.3) vs. 3.2 (2.8–4.1)	0.77 (0.61–0.96) *p =* 0.0184	27 (21–32) vs. 22 (16–30)		negative
RAINFALL	III	Ram + XP or FP vs. Placebo + XP or FP	1L	326 (Ram) vs. 319 (PBO)	PFS	11.2 (9.9–11.9) vs. 10.7 (9.5–11.9)	0.96 (0.80–1.16) *p* = 0.68	5.7 (5.5–6.5) vs. 5.4 (4.5–5.7)	0.75 (0.61–0.94) *p* = 0.011	41 (36–46) vs. 36 (31–42)	*p* = 0.17	positive
RAINSTORM	rII	Ram + SOX vs. Placebo + SOX	1L	96 (Ram) vs. 93 (PBO)	PFS	14.7 vs. 14.3	1.11 (80% CI 0.89–1.40) *p* = 0.55	6.3 vs. 6.7	1.07 (80% CI 0.86–1.33) *p* = 0.70	58 vs. 50	1.37 (80% CI; 0.84–2.24) *p* = 0.40	negative
JVBT	rII	Ram + mFOLFOX6 vs. Placebo + mFOLFOX6	1L	84 (Ram) vs. 84 (PBO)	PFS	11.7 vs. 11.5	1.08 (95% CI 0.73–1.58) *p* = 0.712	6.4 vs. 6.7	0.98 (95% CI 0.69–1.37) *p* = 0.886	45.2 (34.3–56.5) vs. 46.4 (35.5–57.6)	*p* = 0.830	negative
Bando H, et al.	II	Ram + nab-PTX	2L	45	ORR	NR		7.6 (5.4–8.1)		54.8 (41.0–68.0)		positive
RAMIRIS	II	Ram +FOLFIRI vs. Ram +PTX	2L	72 (FOLFIRI) vs. 38 (PTX)	OS at 6 m	6.8 (5.1–11.1) vs. 7.6 (6.1–11.5)	0.97 (95% CI 0.62–1.52)	3.9 (2.8–6.8) vs. 3.7 (2.1–5.5)	0.73 (95% CI 0.48–1.11)	22 vs. 11		negative
HGCSG1603	II	Ram + IRI	2L	35	PFS at 6 m	9.6 (6.4–16.6)		4.2 (2.5–5.4)		25.9 (11.1–36.3)		negative
Kawazoe A, et al.	II	Ram + FTD/TPI (Cohort A/B)	2L/≥3L	33 (A)/31 (B)	DCR			A; 5.9 (4.2–7.9) B; 5.3 (2.8–6.0)		DCR 85 (68–95)/77 (59–90)		positive
**Apatinib/Regorafenib**												
Li J, et al.	rII	Placebo (Group A) Apatinib 850mg (Group B) Apatinib 425mg (Group C)	≥3L	46 (A) 47 (B) 48 (C)	PFS	A; 2.50 (1.87–3.70) B; 4.83 (4.03–5.97) C; 4.27 (3.83–4.77)	B vs. A 0.37 (0.22–0.62) *p* = 0.0017 C vs. A 0.41 (0.24–0.72) *p* = 0.119	A; 1.40 (1.20–1.83) B; 3.67 (2.17–6.80) C; 3.20 (2.37–4.53)	B vs. A 0.18 (0.10–0.34) *p* < 0.001 C vs. A 0.21 (0.11–0.38) *p* < 0.001	A; 0.00 (0.0–7.4) B; 6.38 (1.3–17.5) C; 13.04 (4.9–26.3)		positive
Li J, et al.	III	Apatinib vs. BSC	≥3L	181 (RAM) vs. 92 (PBO)	OS/PFS	6.5 (4.8–7.6) vs. 4.7 (3.6–5.4)	0.709 (0.537–0.937), *p* = 0.0156	2.6 (2.0–2.9) vs. 1.8 (1.4–1.9)	0.444 (0.331–0.595) *p* < 0.001	2.84 (investigator) vs. 0.00 (investigator)	*p* = 0.1695	positive
INTEGRATE	rII	Regorafenib vs. BSC	≥2L	100 (Rego) vs. 52 (PBO)	PFS	5.8 (4.4–6.8) vs. 4.5 (3.4–5.2)	0.74 (0.51–1.08), *p* = 0.147	2.6 (1.8–3.1) vs. 0.9 (0.9–0.9)	0.40 (0.28–0.59) *p* < 0.001			positive

No; number, CI; confidence interval, MST; median survival time, HR; hazard ratio, mPFS; median progression-free survival, ORR; objective response rate, OR; odds ratio, Bev; bevacizumab, IRI; irinotecan, CDDP; cisplatin, TTP; time to progressin, DTX; docetaxel, L-OHP; oxaliplatin, PBO; placebo, OS; overall survival, mDCF; modified DCF, IQR; inerquartile range, PTX; paclitaxel, DCR; disease control rate.

**Table 2:** Clinical trials in combination with immunotherapy.

Trial Name/Authors	Ph	Antiangiogenesis Agent	Immunotherapy	Chemotherapy	Line	No. of Patients	MST (95% CI) (Months)	mPFS (95% CI) (Months)	ORR (95% CI) (%)
JVDF	I	Ramucirumab	Pembrolizumab	none	1L	28	14.6 (5.4–27.7)	5.6 (3.9–12.3)	25 (10.7–44.9)
EPOC1706	II	Lenvatinib	Pembrolizumab	none	1L/2L	29	NR (11.8–NR)	7.1 (5.4–13.7)	69 (49–85)
NivoRam	I/II	Ramucirumab	Nivolumab	none	2L	46	9	2.9	26.7
Xu J et al.	I	Apatinib	SHR1210	none	2L	23	11.4 (8.6–NR)	2.9 (2.5–4.2)	17.4 (5.0–38.9)
Nakajima T, et al.	I/II	Ramucirumab	Nivolumab	Paclitaxel	2L	43	13.1 (8.0–16.6)	5.1 (4.5–6.5)	37.2 (23.0–53.5)
JVDJ	I	Ramucirumab	Duruvalumab	none	≥2L	29	12.4 (5.5–16.9)	2.6 (1.5–7.1)	21
REGOMUNE	II	Regorafenib	Avelumab	none	≥2L	49	7.5 (4.5–15.7)	1.9 (1.8–3.5)	16.7
JVDF	I	Ramucirumab	Pembrolizumab	none	≥2L	41	5.9 (4.4–10.6)	2.5 (1.5–4.2)	7 (1.5–19.9)
REGONIVO	I	Regorafenib	Nivolumab	none	≥3L	25	12.3 (5.3–NR)	5.6 (2.7–10.4)	44 (24.4–65.1)

Ph; phase, No; number, CI; Confidence interval, MST; median survival time, mPFS; median progression-free survival, ORR; objective response rate.

**Table 3:** Ongoing cinical trials of anti-angiogenic therapy in combination with immunotherapy.

Trial Name	Ph	Anti-Angiogenic Agent	Immunotherapy	Chemotherapy	Line	No. of Patients	Primary Endpoint	ClinicalTrials.gov Identifier
IMMUNOGAST	II	Bevacizumab	Atezolizumab	none	≥2L	60	ORR	NCT04739202
2020-03-115	II	Ramucirumab	Pembrolizumab	none	2L/3L	35	ORR	NCT04632459
SEQUEL	rII	Ramucirumab	Pembrolizumab	Paclitaxel	2L	58	ORR	NCT04069273
AIO-STO-0218	I	Ramucirumab	Avelumab	Paclitaxel	2L	59	OS at 6 months	NCT03966118
AHQU-2022001	II	Apatinib	Sintilimab	SOX	1L	31	ORR	NCT05216237
FDZL-CAP	rII	Apatinib	Camrelizumab	none	3L	102	PFS	NCT05095636
MA-GC-II-011	II	Apatinib	Camrelizumab	none	≥2L	20	ORR	NCT04948125
PD-1-APTN-II-AFPGC	II	Apatinib	Camrelizumab	none	≥2L	30	ORR	NCT04006821
NFEC-2020-024	II	Apatinib	Camrelizumab	Irinotecan	2L	85	OS	NCT04934618
HCCSC G05		Apatinib	Camrelizumab	S1 + Docetaxel	1L	35	PFS	NCT04781686
INTEGRATEIIb	rIII	Regorafenib	Nivolumab	none	≥3L	450	OS	NCT04879368
17-00626	II	Lenvatinib	Pembrolizumab	none	≥2L	24	ORR	NCT03321630
NCI-2021-09188		Lenvatinib	Pembrolizumab	none	≥2L	15	ORR	NCT05041153
LEAP-015		Lenvatinib	Pembrolizumab	CAPOX/FOLFOX	1	890	OS (Part 2)	NCT04662710

Ph; phase, No; number, ORR; objective response rate, OS; overall survival, PFS; progression-free survival, r; randomized, SOX; S1 plus oxalliplatin, CAPOX; Capecitabine plus oxaliplatin, FOLFOX; fluorouracil, leucovorin plus oxaliplatin.

## Data Availability

Not applicable.
